# A New Microsphere-Based Immunoassay for Measuring the Activity of Transcription Factors

**DOI:** 10.1007/s12575-010-9030-z

**Published:** 2010-04-14

**Authors:** Yu-Ling Lin, Yun-Ju Lai, Nu-Man Tsai, Tai-Chu Peng, Yen-Ku Liu, Ru-Ping Lee, Chueh-Jen Tsai, Kuang-Wen Liao

**Affiliations:** 1 Institute of Molecular Medicine and Bioengineering, National Chiao Tung University, Hsinchu, Taiwan, Republic of China; 2 School of Medical Laboratory and Biotechnology, Chung Shan Medical University, Taichung, Taiwan, Republic of China; 3 Department of Nursing, Tzu Chi College of Technology, Hualien, Taiwan, Republic of China; 4 Department of Nursing, Tzu Chi University, Hualien, Taiwan, Republic of China; 5 Department of Family Medicine, Hualien Hospital, Department of Health, Executive Yuan, Hualien, Taiwan, Republic of China; 6 Department of Biological Science and Technology, National Chiao Tung University, 300 Room 205 Zhu-Ming Building, 75 Bo-Ai Street, Hsin-Chu, Taiwan, Republic of China

**Keywords:** transcription factor, microsphere-based immunoassay, NF-κB, HIF-1

## Abstract

There are several traditional and well-developed methods for analyzing the activity of transcription factors, such as EMSA, enzyme-linked immunosorbent assay, and reporter gene activity assays. All of these methods have their own distinct disadvantages, but none can analyze the changes in transcription factors in the few cells that are cultured in the wells of 96-well titer plates. Thus, a new microsphere-based immunoassay to measure the activity of transcription factors (MIA-TF) was developed. In MIA-TF, NeutrAvidin-labeled microspheres were used as the solid phase to capture biotin-labeled double-strand DNA fragments which contain certain transcription factor binding elements. The activity of transcription factors was detected by immunoassay using a transcription factor-specific antibody to monitor the binding with the DNA probe. Next, analysis was performed by flow cytometry. The targets hypoxia-inducible factor-1α (HIF-1α) and nuclear factor-kappa B (NF-κB) were applied and detected in this MIA-TF method; the results that we obtained demonstrated that this method could be used to monitor the changes of NF-κB or HIF within 50 or 100 ng of nuclear extract. Furthermore, MIA-TF could detect the changes in NF-κB or HIF in cells that were cultured in wells of a 96-well plate without purification of the nuclear protein, an important consideration for applying this method to high-throughput assays in the future. The development of MIA-TF would support further progress in clinical analysis and drug screening systems. Overall, MIA-TF is a method with high potential to detect the activity of transcription factors.

## 1 Introduction

Transcription factors (TFs) are nuclear proteins that bind to the elements upstream of promoter genes, thereby regulating gene expression [1]. Thus, aberrant activity of TFs (such as oncogenic transcription factors) may lead to a broad range of dysfunctions of human and animal cells, causing diseases such as cancers [2,3]. Therefore, TFs are potential cellular bioindicators for medical diagnosis and targets for drug development [4]. Examples of such important TFs are nuclear factor-kappa B (NF-κB) and hypoxia-inducible factor-1 (HIF-1).

To analyze the potential activation of transcription factors in tumors, it is necessary to develop a simple method to measure the DNA binding capacity of target proteins. Although Western blotting is a good method to detect the content of specific proteins, it can only provide information regarding the total number of the target TFs. Western blotting cannot be used to distinguish between active or inactive TFs [5]. The activity of transcription factors are not always correlated with the TF amounts present in the cells; only the active TFs bound to the transcription factor binding site represent instances of gene expression [6]. Electrophoresis mobility shift assay (EMSA) is the current method used to detect the activity of TFs. Essentially, dsDNA probes containing the TF binding sequences are labeled with the [^32^P]-radioisotope, and the activity of TFs is determined after electrophoresis based on radioactivity levels. In addition, non-radioactive EMSA has been developed, but it is limited by high background and long detection time [5]. Although EMSA can be used to measure the activity of TFs, the devices required and steps of the EMSA procedure do not provide possibilities to develop a high-throughput assay. Although modified enzyme-linked immunosorbent assay (ELISA) assays were developed for measuring the activity of transcription factors in nuclear proteins [7], the procedures for extracting nuclear proteins hindered the application of a high-throughput platform. A reporter gene assay, using transfection of plasmids that contain a mini-promoter with several copies of TF binding elements followed by reporter genes (such as luciferase and GFP), can also be used to determine the activity of TFs in cultured cells. The transfectants generated by reporter plasmids can be used to detect the changes in the activity of TFs after treatment with drugs. Therefore, this technique is suitable for use in high-throughput assays for new drug discovery. However, the efficiency of transfection of variant cells is unreliable and ranges from susceptible to resistant. This variable efficiency may lead to false results when the activity of TFs is compared between different cells.

**Figure 1:**
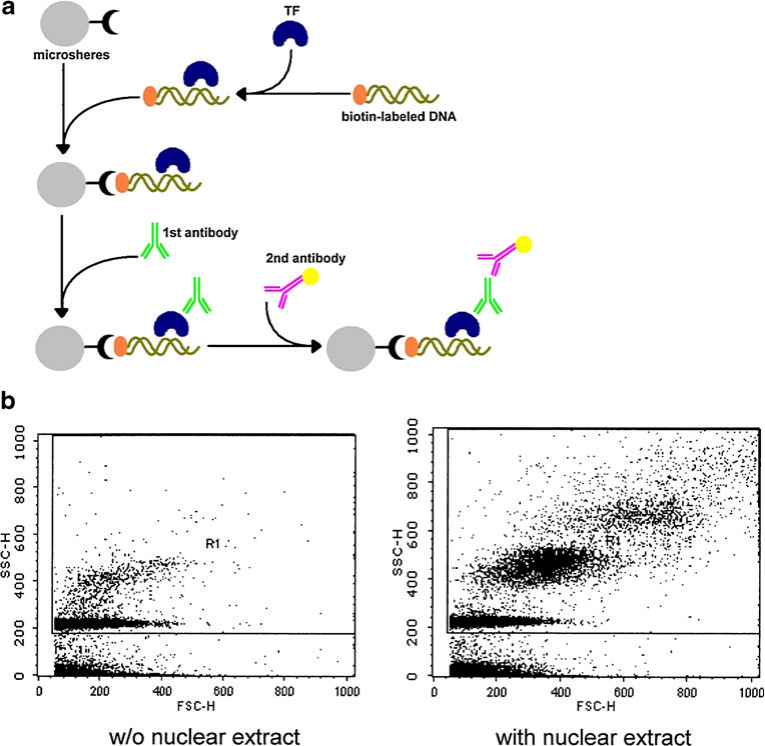
**Experimental design of a microsphere-based immunoassay for measuring the activity of transcription factors (*MIA-TF*)**. **a** Flowchart of MIA-TF. The biotin-labeled dsDNA probes were made to react with TFs in a nuclear extract of the cells. Subsequently, NeutrAvidin microspheres were added into the reactions. After incubation, the mixtures were probed with anti-transcription factor antibody and detected with a fluorescent secondary antibody by flow cytometry. **b** Dot plot (SSC vs. FSC) of the MIA-TF method. The DNA-bound TFs were detected by an immunoassay using a transcription factor-specific antibody and then analyzed using flow cytometry. Microspheres not incubated with nuclear extract were used as negative controls (*left*) to gate the range of effective signals (*R1*). The microspheres incubated with the nuclear extracts increased the fluorescence values in the range of effective signals (*right*). *bio-DNA* biotin-labeled dsDNA, *TF* transcription factor.

NF-κB is a transcription factor that controls the expression of genes involved in cell proliferation, cycle progression, invasion, angiogenesis, metastasis, apoptosis suppression, multiple drug resistance, and inflammatory response in tumor cells [8]. HIF-1 is another TF that regulates gene expression in response to hypoxia and is involved in angiogenesis/vascular remodeling, erythropoiesis, glucose transport, glycolysis, iron transport, and cell proliferation/survival [9,10]. These two TFs were our targets to validate the method we developed to measure the activity of TFs in cells. In this study, NeutrAvidin-labeled microspheres were used as the solid phase to capture the biotin-labeled double-stranded DNA with TF responded elements on it. The flowchart in Figure 1a depicts the strategy to probe the binding activity of transcription factors using this modified microsphere-based immunoassay. The biotin-labeled dsDNA probes were first made to react with a nuclear extract of the cells. Later, subsequent to blocking with polyethylene glycol (PEG), NeutroAvidin microspheres were added to the reactions. After incubation, the mixtures were probed with an anti-transcription factor antibody, washed by centrifugation, detected with a fluorescent secondary antibody, and washed again. Finally, the microspheres were analyzed using flow cytometry. In addition to analyzing the nuclear extract, MIA-TF can be also used to analyze the activity of TFs from total cell lysates of cells cultured in a 96-well culture plate. This may lead to the application of MIA-TF as a high-throughput assay to enable further progress in clinical analysis and drug screening systems.

## 2 Material and Methods

### 2.1 Antibodies and Reagents

Rabbit anti-human NF-κB p50 antibody was purchased from Santa Cruz Biotechnology, Inc. (Santa Cruz, CA, USA). Fluorescein isothiocyanate (FITC)-conjugated goat anti-rabbit antibody was purchased from Jackson ImmunoResearch Laboratories, Inc. PA, USA. Goat anti-mouse FITC-labeled antibody was purchased from Chemicon International, Inc. (Temecula, CA, USA). Mouse anti-human HIF-1α monoclonal antibody was purchased from R&D systems, Inc. (Minneapolis, Minnesota, USA). Deferoxamine mesylate salt (DFO) and hydroquinone were purchased from Sigma Corporation (St Louis, MO, USA). FluoSpheres NeutrAvidin™-labeled microspheres were purchased from Invitrogen (Carlsbad, California, USA).

### 2.2 DNA Probe Preparation

Five types of double-stranded oligonucleotides were used in this study. These were biotin-labeled double-stranded DNA (dsDNA) containing seven times HIF binding site (HBS), biotin-labeled dsDNA containing five times NF-κB binding site (NBS), non-biotin-labeled HBS (cHBS), non-biotin-labeled NBS (cNBS), and non-biotin-labeled dsDNA VP16 without HBS and NBS (VP16). The HBS probe and cHBS probe were prepared by PCR using the pCRII-C2-9 vector which was constructed by our lab and the following primer pairs: 5'-Biotin-GCATCAAGCTTGGTACCG-3' as the forward primer and 5'-AGCTATCGATATCTGCAGAATTCGG-3' as the reverse primer for the HBS probe; 5'-GCATCAAGCTTGGTACCG-3' as the forward primer and 5'-AGCTATCGATATCTGCAGAATTCGG-3' as the reverse primer for the cHBS probe. The NBS and cNBS probes were prepared by PCR using the pNF-κB-hrGFP vector (Stratagene Corporation, Cedar Creek, TX, USA) and the following primer pairs: 5'-Biotin-TCATGTCTGGATCCAAGCTA-3' as the forward primer and 5'-CCGGGGATCCATCTA GATTACCCTCTAGAGTCT-3' as the reverse primer for the NBS probe; 5'-TCATGTCTGGATCCAAGCTA-3' as the forward primer and 5'-CCGGGGATCCATCTA GATTACCCTCTAGAGTCT-3' as the reverse primer for the cNBS probe. The VP16 probe was prepared by PCR using the pCMV-ampR-linker-VP16 vector which was constructed by our lab and the primer pair below: 5'-TATCCTGCAGTCCGCGTACAGCCGCGCG-3' as the forward primer and 5'-TGACCTCGAGCTACCCACCGTACTCGTCAA-3' as the reverse primer for VP16 probe. The sequences of DNA probes are given below:

HBS probe:

Biotin-gcatcaagcttggtaccgagctcggatccactagtaacggccgccagtgtgctggaattcggcttgtt ggagtg**tacgtg**tgtgctcccccaggcattggttgttggagtg**tacgtg**tgtgctcccccaggcatggttgttgg agtg**tacgtg**tgtgctccccaggcatggttgttggagtg**tacgtg**tgtgctccccaggcatggttgttggagtg **tacgtg**tgtgctcccccaggcatggttgttggagtg**tacgtg**tgtgctccccaggcatggttgttggagtg**tacgtg**t gtgctcccccagacgtatatacgtatataagccgaattctgcagatatcgatagct (the bold sequences indicate the HIF binding sites).

NBS probe:

Biotin-tcatgtctggatccaagctag**gggact**ttccgcttg**gggact**ttccgctg**gggact**ttccgctg**gggact**ttccg ctg**gggact**ttccgcggagactctagagggtatataatggatccccgg (the bold sequences indicate the NF-κB binding sites).

VP16 probe:

Tatcctgcagtccgcgtacagccgcgcgcgtacgaaaaacaattacgggtctaccatcgagggcctgctcgatctcccggacgacgacgcccccgaagaggcggggctggcggctccgcgcctgtcctctccccgcgggacacacgcgcagactgtcgacggcccccccgaccgatgtcagcctgggggacgagctccacttagacggcgaggacgtggcgatggcgcatgccgacgcgctagacgatttcgacggacatgttgggggacggggattccccgggtccgggatttaccccccacgactccgccccctacggcgctctggatatggccgacttcgagtttgagcagatgtttaccgatgcccttggaattgcggtacggtgggtagctcgaggtca.

The cHBS and cNBS probes were used for a specific competition test, and VP16 was used for a nonspecific competition test.

### 2.3 Cell Culture

The human cervical cancer HeLa cell line was purchased from the American Type Culture Collection (Manassas, VA, USA). The HeLa cells were cultured in Dulbecco's modified Eagle medium (DMEM) supplemented with 10% fetal bovine serum, 100 U/ml of penicillin/streptomycin, and 10% (*w*/*v*) of sodium hydrocarbonate. The cells were incubated at 37°C in a humidified atmosphere of 5% CO_2_.

### 2.4 Drug Treatment

The iron-chelator desferrioxamine (DFO) induces HIF-1α in normoxia. DFO prevents HIF-1α from proteolysis by inhibiting the activity of iron-dependent prolyl hydroxylases [11]. To activate HIF-1, HeLa cells were seeded (1 × 10^5^ cells per well) in a 24-well plate and cultured in DMEM for 8 h. Cells were treated with 100 μM of DFO in triplicate and then incubated for 16 h at 37°C. Hydroquinone, a reactive metabolite of benzene, is known to inhibit NF-κB activity. The cells were also treated with 25 μM of hydroquinone in triplicate and then incubated for 16 h at 37°C. After the medium was aspirated from the wells, the cells were rinsed with phosphate-buffered saline (PBS) and lysed with lysis buffer (10 mM 4-(2-hydroxyethyl)-1-piperazineethanesulfonic acid (HEPES), 1.5 mM magnesium chloride (MgCl_2_), 10 mM potassium chloride (KCl), 1 mM DTT, 10% (*v*/*v*) protease inhibitor cocktail, and 0.1% (*v*/*v*) Nonidet P-40).

### 2.5 Nuclear Extract

After incubation with or without the target drugs, the HeLa cells were scraped into DMEM medium, washed in cold PBS, and centrifuged at 500 × *g* for 5 min. Next, HeLa cells at a concentration of 2.5 × 10^7^ cells/ml were lysed in lysis buffer (10 mM HEPES), 1.5 mM MgCl_2_, 10 mM KCl, 1 mM DTT, 10% (*v*/*v*) protease inhibitor cocktail, and 0.1% (*v*/*v*) Nonidet P-40) by pipetting up and down. The lysed cells were centrifuged in a microcentrifuge tube at 16,000 × *g* for 5 min at 4°C, and the cytosolic supernatant was removed. The nuclear pellet, consisting of 1 × 10^8^ cells/ml, was dissolved in extraction buffer (20 mM HEPES, 1.5 mM MgCl_2_, 0.42 M NaCl, 0.2 mM EDTA, 25% (*v*/*v*) glycerol, 1 mM DTT, and 1% (*v*/*v*) protease inhibitor cocktail), incubated for 30 min on ice, and centrifuged at 16,000 × *g* for 5 min [12]. The supernatants containing nuclear proteins were collected and stored at -80°C. The protein concentrations were determined by the Coomassie Plus (Bradford method) protein assay reagent (Pierce, Oud-Beijerland, the Netherlands) according to the manufacturer's instructions.

### 2.6 Development of the MIA-TF System

Exactly 1.5 × 10^-11^ nmol of FluoSpheres NeutrAvidin™-labeled microspheres were mixed with PEG to a final concentration of 25 mM to block the microspheres in each reaction. After incubation at room temperature for 30 min, the blocked microspheres were diluted to 100 μl with bovine serum albumin (BSA) buffer (1% BSA in PBS) and centrifuged at 9,300 × *g* for 5 min. The supernatant was decanted and discarded; the pellet was then resuspended with 10 μl PBS.

Biotin-labeled dsDNA (1.5 × 10^-8^ nmol), prepared as described in the DNA probe preparation section, was added to an appropriate concentration of nuclear extract. The final volume of the reaction was adjusted with extraction buffer to 30 μl and incubated at room temperature for 30 min. The same amount of non-biotin-labeled dsDNA was added at the same step. After 30 min of incubation, the prepared microspheres were added to each sample and shaken gently for 15 min at room temperature. The sample was washed with 500 μl of washing buffer (0.02% Tween-20 in PBS) and then centrifuged at 9,300 × *g* for 5 min. After carefully discarding the supernatant, 40 μl of the primary antibody was added to each tube and incubated at room temperature for 1 h. After centrifugation at 9,300 × *g* for 5 min, the supernatant was discarded. Next, 500 μl of washing buffer was added into the sample, and the sample was centrifuged once more to remove the supernatant. Then, 40 μl of the secondary antibody (2.5 μg/ml in 1% BSA buffer) was added to each tube and incubated at room temperature for another 1 h. After the washing steps, the sample was resuspended in 500 μl BSA buffer and was ready for flow cytometry (Becton Dickinson, Mountain View, CA, USA). The parameters for the measurement of fluorescence intensity were as follows:

**Table:** 

Param	Detector	Voltage	Amp/Gain	Mode
P1	FSC	E01	7.00	Lin
P2	SSC	545	1.00	Lin

### 2.7 Whole Cell Extract

HeLa cells were seeded (3,500 cells per well) in a 96-well plate. After incubation for 24 h, cells were scraped in medium, washed in cold PBS, and centrifuged at 500 × *g* for 5 min. The cells were extracted in 50 μl of whole cell extraction buffer (20 mM HEPES, 20% (*v*/*v*) glycerol, 1% (*v*/*v*) Nonidet P-40, 1 mM MgCl_2_, 0.5 mM EGTA, 1 mM phenylmethylsulfonyl fluoride, 1 mM DTT, and 10% (*v*/*v*) protease inhibitor cocktail) by pipetting up and down and incubated on ice for 20 min. After centrifugation at 3,700 rpm (Swing-bucket rotor A-2-DWP, Eppendorf, Hamburg, Germany) for 5 min, the whole cell extract was in the supernatant, the sample was analyzed by modified-MIA-TF after the protein concentrations of the whole cell extracts were determined by the Coomassie Plus (Bradford method) protein assay reagent (Pierce, Oud-Beijerland, the Netherlands) as specified in the instruction manual.

### 2.8 MIA-TF Directly Detect Transcription Factor in 96-Well Plates

Each well of 96-well microtiter plates was blocked with 300 μl of PBS buffer containing 5% BSA (BSA buffer) at room temperature for 1–2 h. Then, the BSA buffer in each well was discarded and the plate was washed three times with PBST buffer (0.05% Tween-20 in PBS buffer). MIA-TF was performed in the prepared 96-well microtiter plate. Briefly, the whole cell lysate was transferred into the wells of the blocked 96-well microtiter plate, and the blocked microspheres were added and mixed gently for 15 min. After centrifugation at 3,500 rpm for 5 min, the supernatant was carefully discarded and 40 μl of the primary antibody was added into each well and allowed to incubate at room temperature for 1 h. The wash procedure described above was repeated using centrifugation; 40 μl of the secondary antibody (2.5 μg/ml in 1% BSA buffer) was then added to each well and incubated at room temperature for 1 h. The samples were washed, resuspended in 200 μl BSA buffer, and finally their fluorescent activity was determined by flow cytometry.

### 2.9 Statistical Analysis

Data were analyzed using the SAS statistical software package (SAS Institute, Inc., Cary, NC, USA). The results were expressed as the mean ± SD. Differences in mean values were evaluated by a one-way ANOVA. The level of significance considered was *p* < 0.05.

## 3 Results

### 3.1 Strategy for the Microsphere-Based Immunoassay for Measuring the Activities of Transcription Factors

The dot plot (forward scatter vs. side scatter) of microspheres without incubation with the nuclear extract was the negative control used to gate the range of effective signals (R1) and to exclude interference in the buffer (Figure 1b). The results indicated that the nuclear extract caused the of SSC and FSC values of the microspheres to increase (Figure 1b). The effects may be due to the binding activity of the DNA binding proteins in nuclear extract.

### 3.2 Determining the Activity of Transcription Factors in Nuclear Extracts by MIA-TF

**Figure 2:**
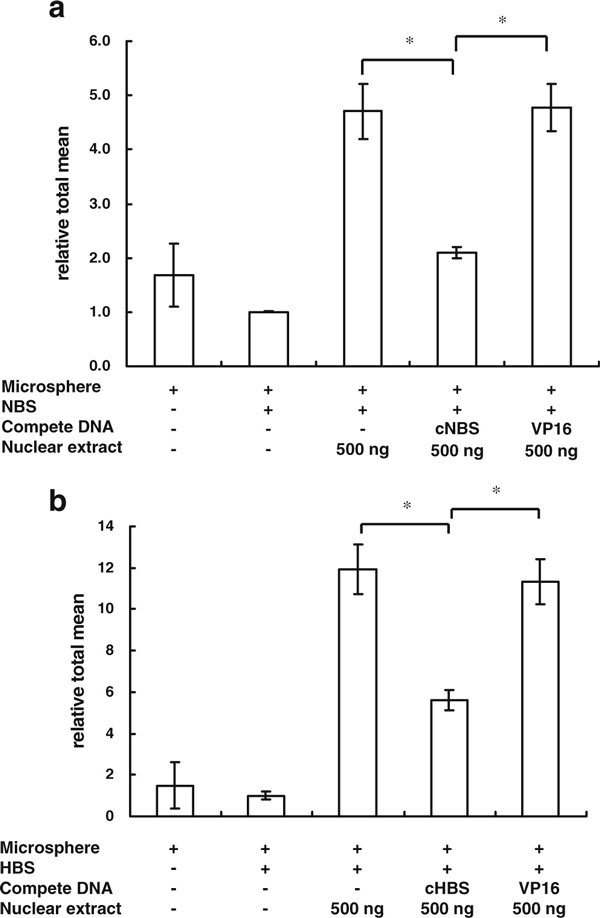
**The MIA-TF method was used to detect the activity of transcription factors**. **a** The MIA-TF method was used to detect the changes in NF-κB expression in 500 ng of nuclear extract. **b** The MIA-TF method detected the changes of HIF-1 expression in 500 ng of nuclear extract. Relative total mean = Events_R1_ × Mean_R1_/Events_R2_ × Mean_R2_. The results are expressed as mean ± SD. *p < 0.05, *n* = 6. *NBS* biotin-labeled dsDNA containing five NF-κB binding site, *cNBS* non-biotin-labeled NBS, *HBS* biotin-labeled dsDNA containing seven HIF binding sites, *cHBS* non-biotin-labeled HBS, *VP16* non-biotin-labeled dsDNA containing no HBS and NBS.

To test whether the MIA-TF method can be used to detect the activity of transcription factors in the nuclear extracts of cells, the NF-κB probe (NBS: dsDNA fragment with biotin-labeled and containing NF-κB binding elements) was first used to detect the NF-κB activity in the nuclear extracts of cells. The results showed that the fluorescence values of microspheres incubated with nuclear extracts were higher than those of microspheres incubated without nuclear extracts for NBS. The increase in fluorescence could be abolished by non-biotin-labeled NBS (cNBS), but was not decreased using unrelated competitive dsDNA fragments with the VP16 binding element (Figure 2a). Another probe, the HIF binding element, HBS, was also used to validate the effects of MIA-TF for the activity of HIF in nuclear extracts. The results also indicated that the HBS probe could be used to detect the activity of HIF as a NBS probe (Figure 2b).

### 3.3 The Sensitivity of MIA-TF

**Figure 3:**
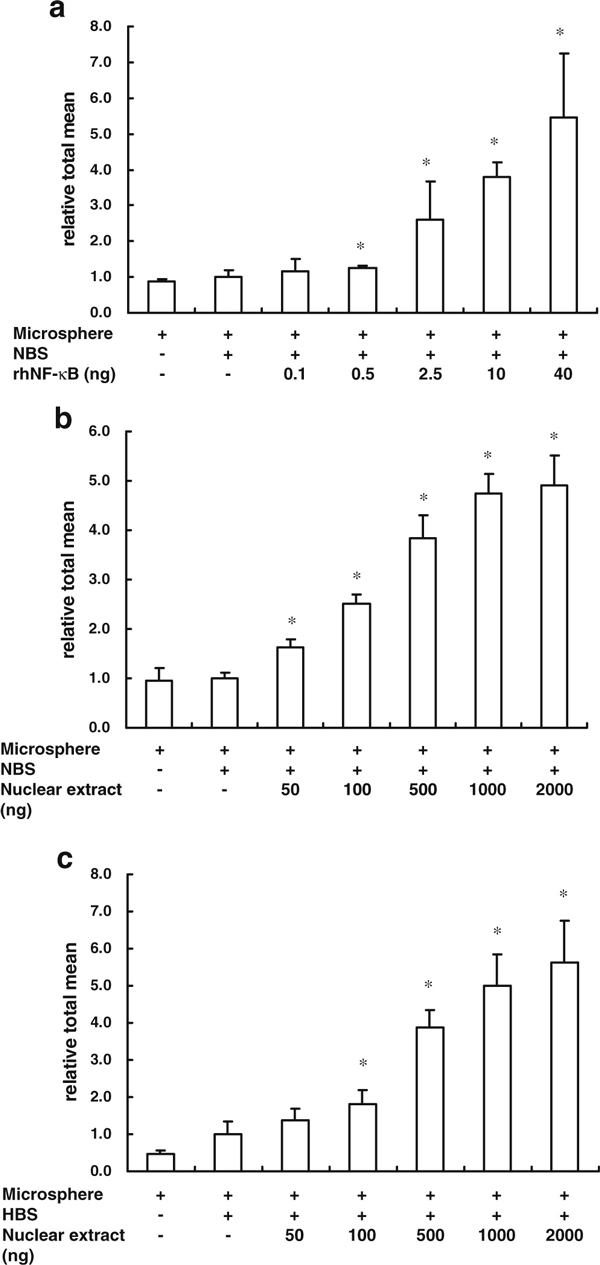
**Sensitivity of MIA-TF detection**. **a** The MIA-TF method was used to detect different contents of recombinant human NF-κB p50 transcription factors. **b** The MIA-TF method was used to detect NF-κB activity in different contents of nuclear extract. **c** The MIA-TF method was used to detect HIF-1 activity in different contents of nuclear extract. The results are expressed as mean ± SD. *p < 0.05 and, compared with the control group (column II), *n* = 6. *NBS* biotin-labeled dsDNA containing five times NF-κB-binding site, *HBS* biotin-labeled dsDNA containing seven times HIF-binding site.

Recombinant human NF-κB proteins (rhNF-κB) were used to determine the sensitivity of the MIA-TF method. The results showed that more than 0.5 ng of rhNF-κB significantly increased the relative fluorescence in a dosage-dependent manner (Figure 3a). Moreover, the sensitivity of the MIA-TF method for NF-κB in the nuclear extracts was also determined, and the results indicate that the MIA-TF method measured the activity of NF-κB in 50 ng of the nuclear extracts. The increases in fluorescence activity were correlated to the amounts of nuclear extracts, which ranged from 50 to 1,000 ng (Figure 3b). In contrast, the HBS probe showed effective sensitivity that ranged from 100 to 1,000 ng for the activity of HIF in the nuclear extracts (Figure 3c).

### 3.4 Determining the Effects of Drugs on the Activity of Transcription Factors Using the MIA-TF Method

**Figure 4:**
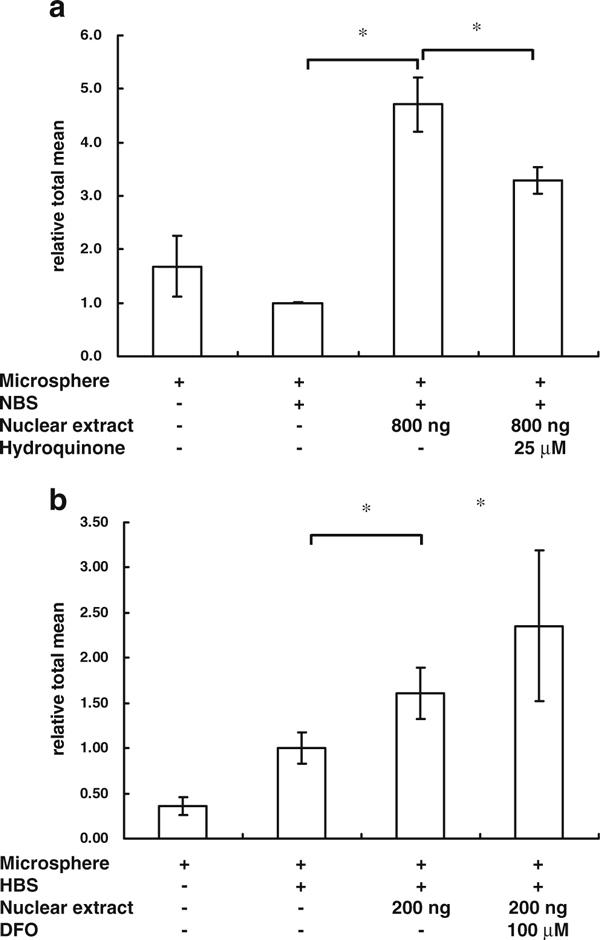
**MIA-TF detected the activity of transcription factors in cells after drug treatment**. **a** After treatment with 25 μM hydroquinone for 16 h, the MIA-TF method was used to detect the changes of NF-κB expression in HeLa cells. **b** After treatment with 100 μM desferrioxamine (*DFO*) for 16 h, the MIA-TF method was also used to detect the changes of HIF-1 expression in HeLa cells. The results are expressed as mean ± SD. *p < 0.05, *n* = 6. *NBS* biotin-labeled dsDNA containing five times NF-κB-binding sites, *HBS* biotin-labeled dsDNA containing seven HIF-binding sites.

Hydroquinone is an inhibitor that suppresses the activity of NF-κB. To monitor whether the MIA-TF method could detect the effects of drugs on the activity of TFs, cells were treated with hydroquinone (25 μM) and the nuclear extracts were assayed. We observed that the suppression of NF-κB activity by hydroquinone was detectable using the MIA-TF assay system (Figure 4a). DFO, an inducer of HIF, was also used to verify the MIA-TF assay for evaluating the effects of drugs on the activity of TFs. The ability of DFO to enhance the activity of HIF was confirmed using the MIA-TF method (Figure 4b).

### 3.5 The Activity of Transcription Factors in Cells were Displayed Using the MIA-TF Method in a 96-Well Culture Plate

The results described above revealed that the MIA-TF method could determine the activity of TFs in nuclear extracts. For application in a high-throughput system, we needed to determine whether the MIA-TF method could be used to analyze the effects of drugs on cells that were cultured in 96-well plates.

**Figure 5:**
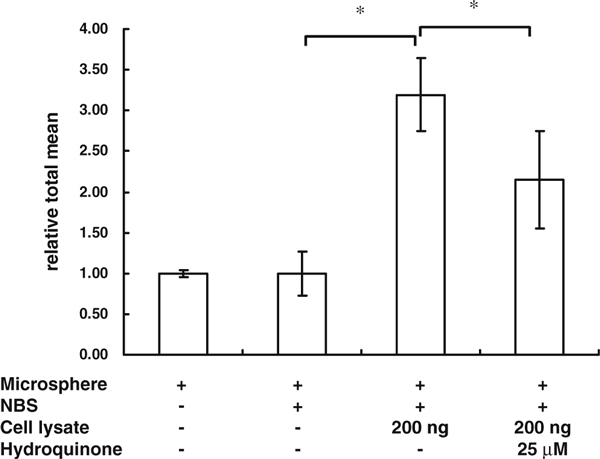
**Modified-MIA-TF directly measured the TF activity in 96-well plates**. The activity of NF-κB was analyzed using a modified-MIA-TF system. After seeding 3,500 HeLa cells/well in 96-well plates, cells were treated with 25 μM hydroquinone for 16 h. The cells were lysed and the activity of transcription factors in cell lysates in 96-well plates was measured. The results are expressed as mean ± SD. *p < 0.05, *n* = 6. *NBS* biotin-labeled dsDNA containing five times NF-κB-binding site.

The results showed that the MIA-TF method could also be used to explore the NF-κB activity of cell lysates in a 96-well plate, even with only 3,500 cells per well (Figure 5). In addition, the reduced fluorescence of the group subjected to hydroquinone treatments were also displayed using the MIA-TF method (Figure 5).

## 4 Discussion

A convenient, sensitive, and specific MIA-TF assay was developed to detect the intracellular activity of TFs. MIA-TF could detect the activity of TFs from 50 ng of nuclear extracts (Figure 2a, b). Because this method provided the fluorescent values in a dosage model, it also determined the precise amount of active TFs in the presence of a TFs standard (Figure 3). Thus, the MIA-TF method may be a good system to determine the degree of tumorigenicity [13] and cellular physiology [14]. Furthermore, this assay could be used to measure the activity of TFs from the cell lysates without extracting nuclear proteins (Figure 5), an important point when considering its application in the discovery of new drugs using a high-throughput platform [15].

The MIA-TF method has several advantageous characteristics, including fast detection, high sensitivity, specific detection, and flexible as well as wide application. Several blocking reagents were tested to circumvent the false positive signals resulting from nonspecific binding of TFs to microspheres; PEG provided the best blocking effect [16]. The 3,500 cells cultured per well in a 96-well plate were directly lysed with cell lysis buffer to obtain about 500 ng whole cellular proteins; the activity of TFs of the latter was directly determined in the well (Figure 5). Although whole nuclear proteins can be detected using ELISA which was created by Jagelska et al. [17], the advantage of the MIA-TF method is that it works without the requirement to purify the nuclear proteins. Furthermore, the MIA-TF method could be conveniently extended to measure any interesting TFs by simply switching the dsDNA probe; it is possible to synthesize short DNA primer pairs with the binding elements of TFs and using specific antibodies.

ELISA-based kits were used for the detection and quantification of TF activation. Both nuclear and whole cell extracts could be assayed using such kits. ELISA has limitations, like the need for a large sample volume, a narrow dynamic range, and complicated dilution procedures [18,19]. No matter which kit is used, the optimal detection of TF activity ranges between 0.5 and 100 μg of nuclear extract. Wang et al. [20] showed that the detection range was between 0.625 and 10 μg of nuclear extract. In our study, the sensitivity of TF activity was quantified as about 0.05–2 μg of nuclear extract. de Jager and Rijkers [21] also indicated that the cytokine detection of bead-based multiplex immunoassays (MIA) was more sensitive than ELISA. Therefore, a three-dimensional solid carrier may be better than the plane-solid carrier to decrease the conformational barriers and raise the sensitivity.

Marligen Biosciences (http://www.marligen.com ) has developed a multiplex TF assay that uses TF probes to determine changes in signaling pathways. The design of the multiplex TF assay is similar to our MIA-TF assay. Theoretically, the multiplex TF assay can be used to detect the activation of different TFs in a single sample by distinguishing between the reactions with different fluorescent microspheres or fluorescence-labeling probes, a useful design for multiple detections of TFs. Like the multiplex TFs assay, the MIA-TF method also can be used to determine the activation of different TFs using a single sample as long as it is combined with antibodies labeled with different fluorescent quantum dots. However, the MIA-TF method has more advantages than the multiplex TF assay. First, the sensitivity of the MIA-TF method was higher: the multiplex TFs assay needs at least 500 ng of nuclear extracts for the detection of TF activity according to the instructions, while the MIA-TF method detected the activity of TFs using 50 ng of nuclear extracts (Figure 3). Although the multiplex TF assay can be used to perform the reactions in a microtiter plate when combined with the nuclear extraction kit, the MIA-TF assay directly measured the activity of TFs from the whole cell lysates in the 96-well microtiter plates. In addition, the multiplex TF assay method highlighted the differences between the TF-bound probe and the unbound probe by the addition of a "proprietary reagent" provided by the company. Without antibody recognition, this proprietary reagent could interfere with the TFs by nonspecific binding of certain DNA binding proteins. In comparison, analysis using the MIA-TF method provided sensitive and reliable detection of the activity of TFs.

## 5 Conclusion

Previously, researchers developing microsphere-based assays used captured antibodies bound to an immobile phase on polystyrene microspheres for multiplexed assays of cytokines, antibodies, hormones, nucleic acids, viruses, and other biomolecules [22,23]. The new MIA-TF method we developed for TF detection shows potential in furthering progress in clinical analysis and drug screening. Overall, the MIA-TF method is a highly promising method for detecting the activity of TFs.
